# Advances in Injectable In Situ-Forming Hydrogels for Intratumoral Treatment

**DOI:** 10.3390/pharmaceutics13111953

**Published:** 2021-11-18

**Authors:** Gi Ru Shin, Hee Eun Kim, Jae Ho Kim, Sangdun Choi, Moon Suk Kim

**Affiliations:** 1 Department of Molecular Science and Technology, Ajou University, 206, World Cup-ro, Yeongtong-gu, Suwon-si 16499, Gyeonggi-do, Korea; mirage1008@ajou.ac.kr (G.R.S.); aasaaaa11@ajou.ac.kr (H.E.K.); jhkim@ajou.ac.kr (J.H.K.); sangdunchoi@ajou.ac.kr (S.C.); 2 Research Institute, Medipolymer, 274-Samsung-ro, Suwon-si 16522, Gyeonggi-do, Korea

**Keywords:** cancer, intratumoral injection, injectable in situ-forming hydrogel, anticancer effect

## Abstract

Chemotherapy has been linked to a variety of severe side effects, and the bioavailability of current chemotherapeutic agents is generally low, which decreases their effectiveness. Therefore, there is an ongoing effort to develop drug delivery systems to increase the bioavailability of these agents and minimize their side effects. Among these, intratumoral injections using in situ-forming hydrogels can improve drugs’ bioavailability and minimize drugs’ accumulation in non-target organs or tissues. This review describes different types of injectable in situ-forming hydrogels and their intratumoral injection for cancer treatment, after which we discuss the antitumor effects of intratumoral injection of drug-loaded hydrogels. This review concludes with perspectives on the future applicability of, and challenges for, the adoption of this drug delivery technology.

## 1. Introduction

Approximately 20% of the world’s population has been diagnosed with some type of cancer, making this one of the most fatal diseases worldwide. Further, the number of cancer patients in 2040 is predicted to increase by almost twice compared with the current estimates [1,2].

Cancer patients are commonly treated by surgery, chemotherapy, immunotherapy, and radiation therapy, as well as targeted therapy, a recently developed therapeutic strategy. Although current therapeutic approaches have been used to successfully treat patients, some of these methods have been linked to various serious side effects and patients often exhibit a cancer recurrence due to metastasis, as well as damage to normal organs [2].

Among the current treatment methods, chemotherapy has been proven to be a highly efficient anticancer therapy. Nevertheless, chemotherapeutic drugs are often non-specific to tumors and can thus affect normal tissues, organs, and the nervous system. Additionally, the bioavailability of these compounds is generally poor [3].

Given the aforementioned challenges and limitations, several studies have sought to improve these side effects and maximize the treatment efficiency of chemotherapeutic drugs. Targeted treatment methods using drug-loaded nanoparticles have recently begun to be actively studied and used in clinical trials [4]. However, certain therapeutic drugs in nanoparticles can accumulate in healthy tissues or organs and cause side effects. Additionally, once nanoparticles are administered in vivo, they can temporarily or permanently remain in normal tissues, and organs and are therefore not suitable for targeted therapy [5,6,7,8]. Therefore, recent studies have developed precise and tumor-specific drug release technologies to minimize the toxicity to normal tissues and organs [9,10,11].

Hydrogels can absorb a large amount of biological media and form a three-dimensional network structure. Therefore, these materials are applicable in a variety of biomedical fields [12]. Hydrogels prepared using biocompatible materials can exhibit excellent biocompatibility and low cytotoxicity. Additionally, these hydrogels can be loaded with various anticancer drugs.

Several studies have recently evaluated injectable in situ-forming hydrogels that exist in liquid form at room temperature but undergo a phase transition when injected into a body [13]. These hydrogels could thus be mixed with anticancer drugs and applied as intratumoral injections that solidify once they enter the body [14,15,16]. Injectable in situ-forming hydrogels could thus eliminate the need for surgical procedures, as they can be intratumorally injected. Thus, injectable in situ-forming hydrogels could provide a far less invasive means to treat cancer patients. For intratumoral injection, injectable anticancer-loaded hydrogel formulations can be easily prepared without anticancer drugs loss and then delivered directly to specific tumor sites, thus minimizing drug accumulation in other organs [17].

There are several types of injectable in situ-forming hydrogels, which are classified based on the mechanisms that mediate their liquid-to-hydrogel transition. These hydrogels can be fabricated via physical bonding (e.g., electrostatic interaction, hydrogen interaction, or hydrophobic interaction) and chemical bonding via covalent bond formation through light, enzymes, and click crosslinking agents [18,19,20].

**Figure 1:**
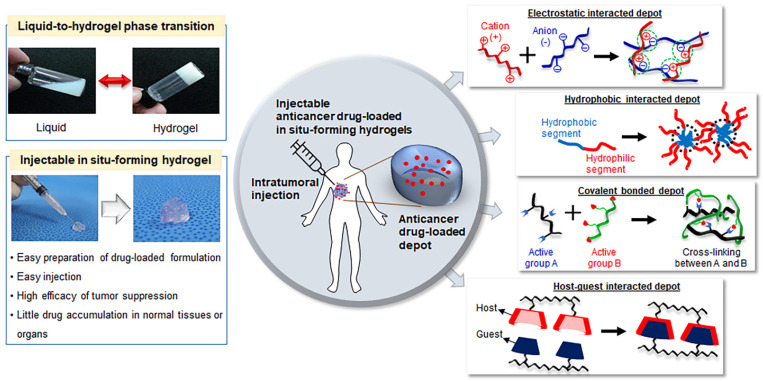
Schematic representation of injectable in situ-forming hydrogel for intratumoral injection (the image was created by G.R.S. and H.E.K. using Adobe Photoshop 7.0).

This review presents a comprehensive and detailed overview of the most recent advances in fabrication strategies for using injectable in situ-forming hydrogels for the intratumoral injection of anticancer drugs (Figure 1). First, this review discusses the preparation of injectable in situ-forming hydrogel formulations via chemical and physical interaction and intratumoral injection in cancer therapy. Afterward, prospective future uses of injectable anticancer drug-loaded hydrogel formulations are proposed.

## 2. Injectable In Situ-Forming Hydrogels

Over the past decades, several studies have described the design and synthesis of several hydrogels for applications in tissue engineering, drug delivery, and bio-nanotechnology. Hydrogels consist of solvent-swelled polymer structures, and water is typically used as the solvent in biological applications. In situ-forming hydrogels can form by swelling in water through different driving forces including the covalent and ionic bonds of hydrophilic polymers [21].

In situ-forming hydrogels offer several advantages in biomedical applications. Specifically, these materials can be prepared in liquid form at room temperature (i.e., outside the human body) and in the absence of trigger materials or mechanisms, after which they can be quickly injected into the body. Furthermore, different hydrogel formulations containing various types of drugs (e.g., anticancer drugs) can be prepared via simple physical mixing. Therefore, in situ-forming hydrogels are promising carrier candidates that could allow for the easy and convenient delivery of anticancer drugs.

For intratumoral injection, the in situ-forming hydrogels not only have to be stable, cost-efficient, and easy to manufacture, but must also be compatible with the drugs to be injected, in addition to promoting a suitable shape formation, uniform and consistent drug loading, and drug release [22,23,24,25]. Several fabrication methods of injectable in situ-forming hydrogels have been reported. This review will focus on injectable hydrogels formed via physical and chemical methods.

### 2.1. Injectable In Situ-Forming Hydrogels Prepared via Physical Interactions

The structure of certain biomaterials relies primarily on physical interactions such as ionic, hydrogen, and hydrophobic bonding, as well as inter- and intra-physical interactions. These physical interactions are relatively weak compared to chemical bonds, thus allowing for the formation of reversible hydrogel matrices according to variations in various biologic conditions [26,27]. The preparation of injectable in situ-forming hydrogels via physical interactions does not require the use of crosslinking agents or chemical modification. Therefore, various biomaterials can be used relatively easily as injectable in situ-forming hydrogels via physical interaction [28,29].

However, given that physical interactions depend on the physical properties of the biomaterial itself, some of the characteristics of these materials are inflexible, including their gelation time, in vitro and in vivo maintenance, and the mechanical properties of the formed hydrogel. Therefore, it may be difficult to accurately control the in vivo performance of these hydrogels [27,28,29]. Nevertheless, these shortcomings can be overcome by using biomaterials with different molecular weights and concentrations, as well as different blends of biomaterials with other components. These procedures can result in the formation of hydrogels with different stiffnesses, viscosities, rheological behaviors, swelling and disintegration behaviors, and biocompatibility. Thus, injectable in situ-forming hydrogels prepared via physical interaction have been widely used for intratumoral injections due to the ease of biomaterial selection and applicability. The different types of injectable in situ-forming hydrogels prepared via physical interaction are described below.

#### 2.1.1. Injectable In Situ-Forming Hydrogels Prepared via Electrostatic Interactions

Electrostatic interaction is one of the most common physical bonds. This type of bond consists of the interaction between two opposite charges such as positive and negative electrolyte groups. These electrostatic interactions can be caused by (poly)electrolyte groups with ionizing or protonating properties, as well as pH changes. These (poly)electrolyte groups can also be affected by external chemical, thermal, and mechanical factors.

Several typical biomaterials contain anionic (poly)electrolytes derived from natural materials such as alginate, chondroitin sulfate, hyaluronate, heparin, sodium carboxymethylcellulose, pectin, dextran sulfate, and xanthan, as well as synthetic materials such as polyacrylic acid derivatives. Cationic (poly)electrolytes include chitosan, polydiallydimethylammonium chloride, spermine, spermidine, polyethylenimine, and polylysine.

Electrostatic interactions can easily be formed between anionic (poly)electrolytes and cationic (poly)electrolytes by instantaneous physical crosslinking, but these interactions can also easily lead to a decreased cross-linking capability with several other (poly)electrolytes in physiological environments [30,31,32]. Materials with anionic (poly)electrolytes (e.g., carboxylates, phosphates, and sulfates) and cationic (poly)electrolytes (e.g., protonated amines) can be individually prepared as injectable (i.e., liquid) formulations before mixing. The (poly)electrolytes in the injectable formulations can induce electrostatic interactions after mixing, resulting in hydrogel formation [33,34,35,36].

However, given the wide variety of electrostatic materials in living organisms, the performance of electrostatic-interaction-based hydrogels may vary depending on the surrounding environments (e.g., in the presence of biological materials and anionic and cationic (poly)electrolytes), as well as the body’s temperature. Resolving these limitations is thus necessary for the further development and widespread adoption of injectable intratumoral hydrogels prepared via electrostatic interactions [29].

#### 2.1.2. Injectable In Situ-Forming Hydrogels Prepared via Hydrophobic Interaction

Under physiological conditions, hydrophobic materials with non-polar groups repel water and aggregate among themselves or with other non-polar materials. In contrast, amphiphilic materials containing both hydrophilic and hydrophobic groups can either dissolve or precipitate in water depending on the environmental conditions. Certain environmental conditions favor the dissolution of hydrophilic structures in water, thus inducing the dissolution of amphiphilic materials in aqueous solution. In other conditions, precipitation can occur via the dehydration of the aqueous solution by the hydrophobic segments [34,37].

Given the difference between body temperature and room temperature, the solubility of amphiphilic materials in water may vary under physiological conditions. This phenomenon can cause amphiphilic materials to undergo a phase transition from their dissolved to their precipitate states.

Various amphiphilic materials have been developed and utilized as injectable in situ-forming hydrogels via hydrophobic interactions. Poly(ethylene glycol) (PEG) is among the most widely studied hydrophilic structures in the biomedical field. Various amphiphilic materials have been developed by incorporating hydrophobic structures into PEG blocks to be used as injectable in situ-forming hydrogels [38]. The developed hydrophobic structures include poly(propylene oxide) (PPO), poly(lactide-co-glycolide) (PLGA), polylactic acid (PLA), poly(ε-caprolactone-co-D,L-lactic acid) (PCLA), polycaprolactone (PCL), poly(trimethylene carbonate) (PTMC), poly(δ-valerolactone), poly (1,4-dioxan-2-one) PDO, PCL-co-PTMC, PCL-co-PDO, polysebacic acid, polyphosphazenes, and poly(N-isopropylacrylamide). A variety of amphiphilic materials composed of hydrophobic and hydrophobic structures can be prepared as liner blocks, dendrimers, and network structures.

These materials possess varying advantages depending on their hydrophilic and hydrophobic group composition, including their adjustable in vivo mechanical properties and in vivo biodegradation [38]. However, amphiphilic materials must be soluble in biological media to serve as carriers for therapeutic agents. Given that many of the developed amphiphilic materials are synthetic, potential challenges such as immune reactions should be comprehensively evaluated prior to their in vivo application [39].

### 2.2. Injectable In Situ-Forming Hydrogels Prepared via Covalent Bonding

A covalent bond is the irreversible linking of one molecule (e.g., a biomaterial chain) to another. The formation of covalent bonds between intra- and inter-biomaterial chains can lead to permanent biomaterial fixing or promote changes in the physical properties of the original biomaterials [40,41]. If the biomaterial is water-soluble, it can form a water-swellable biomaterial network via the covalent bonding between intra- and inter-biomaterial chains, resulting in the formation of injectable in situ-forming hydrogels [42]. Covalent bonding can improve the mechanical properties of injectable in situ-forming hydrogels, thus increasing their resistance to dissolution in aqueous solutions.

Covalent bonds between intra- and inter-biomaterial chains can be formed by chemical reactions that are initiated by heat, pressure, pH changes, or irradiation [43], among which heat application is the most common strategy. Nevertheless, applying heat above body temperature would not be feasible in vivo, and this greatly limits the clinical applicability of injectable in situ-forming hydrogels prepared using heat.

Injectable in situ-forming hydrogels prepared via covalent bonding require functional or crosslinking materials with which to chemically react. Although most functional or crosslinking materials can cause toxicity, injectable hydrogels prepared via covalent bonding have been widely used in intratumoral injections. The sections below describe the types of injectable in situ-forming hydrogels prepared via photo-irradiation, click reaction, and enzyme activity to form covalent bonds.

#### 2.2.1. Injectable In Situ-Forming Hydrogels Prepared via Photo-Irradiation

The photo-reaction method for hydrogel synthesis is generally regarded as fast and practical [26,44]. Additionally, various photosensitive materials have been developed [45] and have been widely studied in a variety of biological fields [46,47,48,49,50]. Among them, azobenzene, spiropyran, nitrobenzyl, galactose, 7-diethylamino-4-thiocoumarinylmethyl, cumarin, and cinnamic acid contain anthracene or acrylate groups (photosensitive chromophores), which can be activated within a few seconds of light irradiation.

Nevertheless, unreacted photosensitive chromophores can cause inflammatory reactions. Additionally, light irradiation (particularly UV light) can lead to cytotoxicity and potential genetic mutations. Furthermore, very few compounds could serve as suitable solvents and non-cytotoxic activators (i.e., photoinitiators) in vivo [51,52]. Instead, biomaterials containing these photosensitive chromophores or acrylate groups can also be easily activated by light irradiation. This can enable facile and rapid hydrogel formation via light irradiation when performing in vivo injections, thus enabling injection position control [53].

#### 2.2.2. Injectable In Situ-Forming Hydrogels Prepared via Click Reaction

Covalently click-cross-linked biomaterials prepared as a solution could be used for the development of injectable in situ-forming hydrogels. Click reactions, formed by the mixing of click-group-modified biomaterials, can rapidly form hydrogels with tunable mechanical properties for in vivo applications [54,55]. In recent years, click reaction materials (e.g., formed via the copper-catalyzed azide–alkyne cycloaddition or alkyne–azide reactions, Diels–Alder reaction, Schiff base formation, Michael addition, or thioenol addition, among others) have been introduced into biomaterials with various structures (e.g., linear, dendrimer, and network).

Click reactions between click reaction materials rapidly form bioorthogonally cross-linked hydrogels without catalysts or external energy in aqueous media, as well as under physiological environments [54,55,56,57]. Individual solutions of click-group-modified biomaterials can easily allow for the covalent formation of injectable in situ-forming hydrogels within a few seconds. However, click reactions are potentially cytotoxic, as they involve the use of copper. Additionally, a click reaction’s time may depend on the regiospecificity of the click reagents [58,59].

### 2.3. Injectable In Situ-Forming Hydrogels Prepared via Enzyme Activity

Enzyme-mediated crosslinking can enable the formation of hydrogels in physiological conditions, and injectable in situ-forming hydrogels prepared via enzymatic reactions are increasingly being used as alternatives to metal catalysts and photo-irradiation [60]. 

Active enzymes for injectable in situ-forming hydrogels include laccases, horseradish peroxidase (HRP), transglutaminases (TGases, protein-glutamine gamma-glutamyltransferase), tyrosinase (Tyr), and lysyl oxidase coupled with hydrogen peroxide (H_2_O_2_) to support the reaction [61,62,63,64,65,66,67,68,69]. Enzymatic-mediated crosslinking using HRP induces the binding of aniline, phenol, and its derivative tyramine in the presence of H_2_O_2_ [70,71,72,73]. Enzymatic-mediated crosslinking forms strong covalent bonding, with reactions occurring in less than 10 min. Additionally, the kinetics of in situ-forming hydrogel synthesis can be manipulated by controlling the enzyme concentration. Further, the products of these reactions tend to be highly biocompatible and are especially well suited to the preparation of injectable hydrogels. Nevertheless, several physicochemical factors such as the pH, temperature, and steric hindrance caused by the substrate structure can significantly affect the catalytic activity of enzymes [74].

## 3. Intratumoral Injection Using Anticancer Drug-Loaded Injectable Hydrogels

**Figure 2:**
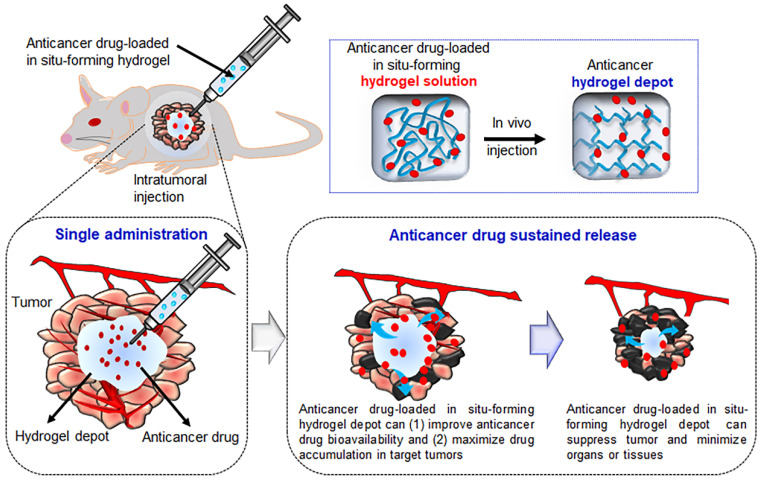
Schematic representation of intratumoral injection of anticancer drug-loaded injectable hydrogels (the image was drawn by G.R.S. and H.E.K. using Adobe Photoshop 7.0).

Intratumoral injection can maximize the efficiency of chemotherapeutic agents and minimize their toxicity to normal organs and tissues other than the target tumor. Additionally, the administration of chemotherapeutic compounds via intratumoral injection could greatly reduce the amount of a drug required for a single administration compared with conventional approaches in which anticancer drugs are repeatedly administered (Figure 2). Therefore, the injectable in situ-forming hydrogels described in the previous sections can easily be prepared as anticancer drug-loaded hydrogel formulations and could be highly effective as a non-invasive method to treat cancer via direct intratumoral injection [75,76].

Table 1 and Table 2 summarize a selection of recent studies that evaluated the applicability of anticancer drug-loaded in situ-forming hydrogels for intratumoral injection, which will be described in the following section.

### 3.1. Intratumoral Injection Using Hydrogels Prepared via Electrostatic Interactions

As discussed above, anticancer drugs can easily be mixed into electrostatic interaction hydrogels prepared using several electrostatic biomaterials.

Huayamares et al. prepared electrostatic interaction hydrogels using glatiramer acetate (GA) and hyaluronic acid (HA) [77]. In a comparative experiment, the electrostatic interacted GA/HA hydrogel was injected into solid tumors using a hydrogel prepared from GA and nonionic PEG. After intratumoral injection, the GA/HA exhibited a higher tumor retention rate than the GA/PEG due to electrostatic interaction, and the drug release rate of these different hydrogels also varied when administered to treat tumor fibrosis.

Chitosan (CS) is enzymatically degradable, generally non-cytotoxic, and biocompatible, and therefore does not cause adverse effects on healthy organs and tissues near the injected tumor site. CS is a cationic polymer with two functional groups, hydroxyl and amine, and an overall positive charge. Thus, the cationic nature of chitosan (CS) enables this biopolymer to electrostatically interact with anionic molecules such as β-glycerophosphate (β-GP). Particularly, a single intratumoral injection of this CS/β-GP system showed good efficacy in mammary-tumor-bearing mice [78,79]

Previous studies sought to increase the efficacy of other drug delivery carriers by taking advantage of the electrostatic interactions with CS. For example, some studies reported on the applicability of a CS/β-GP-based material coupled with carboplatin-loaded PCL nanoparticles and paclitaxel (PTX)-loaded PLGA microparticles. Further, carboxymethylcellulose is an anionic (poly)electrolyte, and therefore hydrogels can be produced via the electrostatic interactions between this compound and chitosan (CCS). Kim et al. prepared curcumin-loaded microspheres to increase the in vivo half-life of curcumin. The authors reported that electrostatically interacting CCS hydrogels with curcumin-loaded microspheres showed good antitumor efficacy in animal models after intratumoral injection [80].

Collectively, these studies demonstrated that electrostatically interacting hydrogels with/without drug-loaded nano- and micro-particles can synergistically enhance the anticancer activity. Although electrostatically interacting hydrogels can easily be formed using anionic and cationic biomaterials, they tend to be sensitive to changes in pH and are thus easily affected by the protonation and deprotonation of anionic and cationic biomaterials [81]. These properties can be a disadvantage; however, pH-responsive electrostatic interacted hydrogels can be prepared via the protonation and deprotonation of ionic biomaterials. Furthermore, electrostatically interacting hydrogels are prone to in vivo degradation due to erosion and cracking, thus impairing their mechanical properties and controlled drug release.

### 3.2. Intratumoral Injection Using Hydrophobically Interacting Hydrogel

As described above, several studies have evaluated the applicability of hydrophobically interacting PEG–polyester block copolymers for the preparation of hydrogels. The most widely used polyesters are PLA, PLGA, and PCL.

Shi et al. used a poly(D,L-lactide)–PEG–poly(D,L-lactide) (PDLLA–PEG–PDLLA) triblock copolymer co-loaded with gemcitabine and cisplatin for a synergistic combination therapy to treat pancreatic cancer [82]. Kim et al. prepared injectable in situ-forming hydrogels using a MPEG–PCL diblock copolymer for intratumoral injection of doxorubicin (DOX), PTX, and 5-fluorouracil (5-Fu) [83,84]. Additionally, the intratumoral injection of PLGA–PEG–PLGA triblock copolymers enabled the sustained release of tamoxifen and Herceptin [85,86].

Recently, OncoGel^TM^ (MacroMed Inc., Salt Lake City, UT, USA) has employed commercial formulations of a PLGA–PEG–PLGA triblock copolymer to deliver PTX to the tumor site in solid malignancies [87]. The efficacy of hydrophobically interacting hydrogels has been preliminarily demonstrated in a Phase IIa study for intratumoral chemotherapy; however, no significant effects on the overall tumor response were observed.

### 3.3. Intratumoral Injection Using Photo-Irradiated Hydrogels

Some hydrogels with photoresponsive agents can be utilized for intratumoral injection. The o-nitrobenzyl or azobenzene groups in PEG- or PEG–polyester-based hydrogels undergo irreversible or reversible reactions when irradiated with UV light, respectively [88,89]. Another study examined the applicability of a hydrogel composed of indocyanine green–alginate (i.e., two near-infrared (NIR)-responsive agents) and Ca^2+^/Mg^2+^ for localized tumor ablation [90]. This injectable indocyanine green–alginate hydrogel has been successfully applied as a highly efficient photothermal therapy in vivo without NIR-induced side effects. Nevertheless, NIR-I light (650–950 nm) penetrates <1 cm into the tissues, and therefore this approach is only suitable to treat superficial and thin tumors. NIR-II light (1000–1700 nm) provides a more feasible means for the treatment of large superficial tumors due to its tissue penetration depth of approximately 3–5 cm; however, its clinical application to the treatment of deep tumors remains restricted [91].

Mukerji et al. developed a photoradiation-controlled intratumoral depot (PRCITD) driven by convection-enhanced delivery (CED) to spatiotemporally control tumors and anticancer drug coverage [92]. This intratumoral depot consisted of a recombinant elastin-like polypeptide (ELP) containing periodic cysteine residues and was conjugated with a photosensitizer [chlorin-e6 (Ce6)] at the N-terminus of the ELP. The photodynamic therapy provided by the PRCITD caused significant tumor inhibition in a Ce6 dose-dependent manner. Additionally, the combination of photodynamic and intratumoral radionuclide therapy co-delivered by the PRCITD provided a greater antitumor effect than either monotherapy alone. These findings suggest that the PRCITD could provide a stable platform for the co-delivery of anticancer drugs to induce synergistic effects.

In intratumoral injection using photo-irradiated hydrogels, increasing the power density (W/cm^2^) and the exposure time to irradiation leads to greater penetration. Given the substantial limitations of UV light (e.g., poor penetration and risk of damaging tissues), approaches involving upconversion of low-energy photons (e.g., near-infrared; NIR) into high-energy photons (e.g., UV) are promising alternatives.

### 3.4. Intratumoral Injection Using Click Crosslinking Hydrogel

Several biomaterials with click crosslinking molecules can be prepared as a solution and have been used to rapidly form injectable hydrogels in physiological environments. The click crosslinking between functionalized molecules has a high efficiency and excellent specificity at high reaction rates. These properties enable the quick and facile formation of intratumoral depots of anticancer drugs after intratumoral injection.

Xu et al. prepared PEG-based dendrimer hydrogels consisting of alkyne dibenzocyclooctyne and PEG-bisazide. Bioorthogonal depots formed via azide–alkyne cycloaddition after intratumoral injection and exhibited a high cytocompatibility, thus enabling the sustained release of the anticancer drug 5-Fu and suppressing the tumor growth [93].

Emoto et al. used an HA-aldehyde and HA-adipic dihydrazide with cisplatin for intratumoral injection. The time of gelation via cross-linking was modified by changing the concentration of the HA-aldehyde and HA-adipic dihydrazide. Click crosslinked cisplatin-loaded HA was successfully implemented to locally deliver cisplatin to live mice [94].

Kim et al. described an in situ-forming PEG hydrogel produced via cross-linking between thiol and maleimide [95]. The gelation time of this material could be controlled from 15 s to 5 min by modifying the thiol and maleimide concentrations. The TRAIL protein-loaded hydrogel quickly formed a depot and exerted anticancer effects on tumors of Mia Paca-2 cell-xenografted BALB mice.

Although click crosslinking can form a stable depot system for the intratumoral delivery of anticancer drugs, the utilized cross-linking agents can be toxic and have poor biocompatibility, which greatly limits the applicability of this approach. Additional studies are thus required to develop non-toxic crosslinking reagents [96].

### 3.5. Intratumoral Injection Using Enzyme-Mediated Crosslinking Hydrogels

Enzyme-reactive hydrogels are typically obtained through enzyme-mediated crosslinking. The preparation of enzyme-reactive hydrogels for intratumoral injection requires the incorporation of an enzyme-specific substrate or a substrate-mimicking material into the hydrogel.

Tang et al. prepared cytarabine HA-tyramine (Ara-HA-Tyr) hydrogel conjugates [97]. The enzyme-reactive hydrogels were formed through the oxidative coupling of tyramines by H_2_O_2_ and HRP. These enzyme-reactive hydrogels exhibited a robust synergistic antitumor efficacy when combined with radiotherapy in the Lewis lung cancer xenograft model.

Oh et al. investigated a gelatin-hydroxyphenyl propionic acid (GHPA)-based hydrogel composed of hydroxyphenyl propionic acid conjugated to gelatin obtained via HRP- and H_2_O_2_-mediated cross-linking [98]. The enzymatic cross-linking reaction of this system can easily be manipulated to achieve hydrogels with desired properties such as gelation time, mechanical stiffness, and degradation rate. More importantly, the authors demonstrated a synergistic antitumor effect and induction of tumor-specific immune responses via the hydrogel-mediated sustained release of oncolytic adenovirus and dendritic cells in solid tumors. 

Xu et al. synthesized an L-phenylalanine-based low-molecular-weight gelator containing thioketal and a control gelator without reactive oxygen species (ROS)-cleavable bonds [99]. Enzyme-reactive hydrogels co-loaded with DOX and a photosensitizer were intratumorally injected into 4T1-breast-tumor-bearing mice and rendered antitumor effects in vivo.

Collectively, the above-described findings demonstrate that enzyme-reactive hydrogels exhibit a high substrate specificity (both regioselectivity and stereoselectivity) and prevent the expensive and time-consuming separation of by-products and intermediates. Nevertheless, enzyme reactive hydrogels have recently been linked to cross-reactivity problems in vivo, thus highlighting the need for further research to enhance the specificity of enzymes to their target substrates [100].

**Table 2:** Intratumoral injection using an injectable hydrogel through covalent bonding with anticancer drugs.

	Hydrogel Materials	Drugs or Agents	Cells	Cancer Type	Ref.
Photo Irradiation	Photothermal Ca^2+^/Mg^2+^ stimuli-responsive ICG–alginate hydrogel	ICG	4T1	Breast cancer cell	[90]
	cE6	cELP [A_14_VC]_16_ gene	FaDu	Squamous cell carcinoma	[92]
Click reaction	DH-P-G4-PDBCO (P-G4-PDBCO + PEG-BA)	5-Fu	HN12	HNSCC	[93]
	HA–ADH, HA-CHO	Cisplatin	MKN45P	Human gastric cancer	[94]
	HAS-SH/PEG-MAL hydrogel	TRAIL protein	Paca-2	-	[95]
Active enzyme	Ara-HA-Tyr hydrogel; H_2_O_2_, HRP	Cytarabine	LLC	Lung cancer	[97]
	GHPA-based hydrogel	Ad, DC	MLLC	-	[98]
	DOX–ZnPCS_4_-coloaded gel	DOX	4T1	Breast cancer cell	[99]

ICG—indocyanine green; cE6—chlorin-e6; cELP—cysteine containing elastin-like polypeptides; FaDu—human squamous cell carcinoma; DH-P-G4 polyamidoamine generarion 4.0 dendrimer hydrogel; PDBCO—PEGylated dibenzocyclooctyne; PEG-BA—polyethylene glycol bisazide; HNSCC—Head and neck squamous cell carcinomas; HA–ADH—Hyaluronic acid–adipic dihydrazide; HA-CHO—Hyaluronic acid aldehyde form; HAS-SH—thiolated human serum albumin; PEG–MAL—4-arm polyethylene glycol–maleimide; TRAIL—TNF-related apoptosis inducing ligand; Ara-HA-Tyr—cytarabine hyaluronic acid-tyramine; HRP—Horseradish peroxide; LLC—Lewis lung cancer; GHPA—gelatin-hydroxyphenyl propionic acid; Ad—oncolytic adenovirus; DC—dentritic cell; MLLC—murine Lewis lung carcinoma cell line.

## 4. Conclusions and Outlook

Research on in situ-forming hydrogels has recently garnered increasing attention, as this technology could enable the localized delivery of anticancer drugs via intratumoral injection. Here, we discussed the preparation of anticancer-drug-loaded injectable in situ-forming hydrogels via chemical and physical interactions for intratumoral injection in cancer therapy. However, this review did not describe the release behavior of anticancer drugs with different lipophilic properties or multiple drugs from hydrogel depots [101,102].

The properties of in situ-forming hydrogels are highly associated with their chemical and physical interactions, and therefore the functional improvement of these materials is extremely dependent on innovation of fabrication strategies. Normally, in situ-forming hydrogels generated via chemical interactions exhibit desirable mechanical properties, whereas in situ-forming hydrogel formulations obtained via physical interactions are more biocompatible due to the absence of chemical crosslinking agents in their composition. Therefore, novel and more comprehensive fabrication strategies for situ-forming hydrogels could pave the way for the development of hydrogel formulations containing various anticancer drugs.

Intratumoral injection technology has recently undergone tremendous progress, including the design of in situ-forming hydrogels optimized for the effective delivery of anticancer drugs. Further, we anticipate that the applications and adoption of this novel drug-delivery technology will continue to grow. The primary objective of intratumoral injection is the effective delivery of anticancer drugs to the tumor site with minimal or no systemic drug bioavailability and no toxicity to healthy organs and tissues. Therefore, in situ-forming hydrogels must enable the sustained release of anticancer drugs from depots after intratumoral injection, thus localizing the anticancer drug exclusively to the tumor site. Future studies on intratumoral injection using in situ-forming hydrogels must address the applicability of this technology to different cancer-affected tissues or organs in clinical environments. 

Although the efficacy of in situ-forming hydrogels for cancer treatment has been repeatedly demonstrated in several proof-of-concept experiments using various animal models and cancer types, translating this technology to clinical applications in humans will pose several important challenges. 

Despite the recent progress in the development of in situ-forming hydrogels for intratumoral injection, several challenges still need to be considered. The first major challenge is the immunogenicity of in situ-forming hydrogel materials to healthy organs and tissues near the injected tumor site, as most injected depots can cause serious inflammation at the injected site. Additionally, in situ-forming hydrogels must be biodegradable under the tumor microenvironment conditions, ideally at a similar rate to the tumor’s decrease. Moreover, the development of intratumoral injection technology requires a thorough understanding of the responses of tumor tissues to in situ-forming hydrogels, as many situ-forming hydrogels are designed and tested in hypothetical conditions and not in the tumor microenvironment. Finally, the spatiotemporal release of anticancer drugs from the depot is another important challenge that must be overcome in order to maximize the efficacy of the hydrogels for the desired treatment period.

Therefore, the following factors must be considered for clinical translation: (1) biomaterials for in situ-forming hydrogels must meet perfect biocompatibility and biodegradability standards; (2) in situ-forming hydrogels must be highly responsive to human tumors; and (3) drug loaded-in situ-forming hydrogels must be evaluated in human trials, paying special attention to the surrounding tumor processes, age, and physical activity.

A more comprehensive understanding of the properties of injectable in situ-forming hydrogels would contribute to the improvement of patients’ convenience while reducing drugs’ systemic toxicity and also allowing for the programmable delivery and sustained release of anticancer drugs from the hydrogel depot—all of which would facilitate clinical translation. In summary, a joint multidisciplinary effort is urgently needed to develop and apply novel strategies that might help to materialize the tremendous potential of injectable in situ-forming hydrogel technology for intratumoral injection. Achieving this would greatly contribute to the advancement of cancer therapy in the near future.

## Figures and Tables

**Table 1:** Intratumoral injection using an injectable hydrogel through physical interaction with anticancer drugs.

	Hydrogel Materials	Drug or Agents	Cells	Cancer Type	Ref.
Electrostatic interactions	HA, GA (HAMA PEGDA) HAMA	Glatiramer acetate	AT-84	Head and neck (HN)	[77]
	CS, β-GP	Carboplatin	B16F1	Melanoma	[78]
	CS, β-GP, PLGA	Paclitaxel	M-234p	Mammary adenocarcinoma	[79]
	CMC, CHI	Cur	A253	-	[80]
	Na-DOC-hydrogel	DOX	MDA-MB-231	Epithelial cell	[81]
Hydrophobic interaction	PDLLA–PEG–PDLLA triblock copolymer	GEM, DDP	BxPc-3	Pancreatic cancer	[82]
	MPEG-b-(PCL-ran-PLLA) diblock copolymer	5-Fu	B16F10	Melanoma	[83]
	MPEG–PCL diblock copolymer	DOX	B16F10	Melanoma	[84]
	PLGA–PEG–PLGA triblock copolymers	Tamoxifen	MCF-7	ERα-positive breast cancer	[85]
	PLGA–PEG–PLGA triblock copolymers	Herceptin	SK-BR-3	HER2+ breast cancer	[86]

HA—hyaluronic acid; GA—glatiramer acetate; HAMA—methacrylated HA; PEGDA—polyethylene glycol diacrylate; CS—chitosan; β-GP—β-glycerophosphate; CMC—caboxylmethycellulose; CHI—chitosan; Cur—curcumin; Na-DOC-hyd—sodium deoxycholate hydrogel; DOX—doxorubicin; PDLLA–PEG–PDLLA—poly(D,L-lactide)–poly(ethylene glycol)–poly(D,L-lactide); GEM—gemcitabine; DDP—cis-platinum;; MPEG-b-(PCL-ran-PLLA)—poly(ethylene glycol)-b-(polycaprolactone-ran-poly-L-lactic acid); 5-Fu—5-fluorouracil; MPEG–PCL—poly(ethylene glycol)-b-polycaprolactone; PLGA-PEG-PLGA—poly(lactic acid-co-glycolic ac-id)-b-poly(ethylene glycol)-b-poly(lactic acid-co-glycolic acid).

## Data Availability

The data presented in this study are available on request from the corresponding author.
